# ﻿Catalog of the genus *Cylindrepomus* Blanchard (Coleoptera, Cerambycidae, Dorcaschematini) in the Philippines, with description of a new species from northern Mindanao

**DOI:** 10.3897/zookeys.1116.86906

**Published:** 2022-08-04

**Authors:** Milton Norman D. Medina, Melbert James G. Baul, Analyn A. Cabras

**Affiliations:** 1 Coleoptera Research Center, University of Mindanao, Davao City, Philippines University of Mindanao Davao City Philippines; 2 Human Resources for Health Network, Department of Health Center for Health Development - Northern Mindanao, J.V. Seriña Street, Carmen, Cagayan de Oro City, Philippines Human Resources for Health Network, Department of Health Center for Health Development - Northern Mindanao Cagayan de Oro City Philippines

**Keywords:** Beetle, conservation, Davao City, Dorcaschematini, Mindanao, Philippines

## Abstract

A catalog of the genus *Cylindrepomus* Blanchard, 1853 in the Philippines, along with the description of a new species from northern Mindanao, is presented. Notes on the ecology, threats, and conservation of the new species are also provided.

## ﻿Introduction

*Cylindrepomus* Blanchard, 1853 is a tropical genus of long-horn beetles (Cerambycidae) (type species *Cylindrepomusnigrofasciatus* Blanchard, 1853) distributed within South China, Southeast Asia, and Oceania. They are easily distinguished from other members of Dorcaschematini for having highly punctate, tomentose elytra covered with recumbent hairs, a bulbous and coarsely granulated scape, and 3^rd^ antennomere 2–4 times longer than scape.

There are 14 species and one subspecies of *Cylindrepomus* in the Philippines; all are endemic to the country, with the majority distributed in a specific island or mountain range. There are four species described from Mindanao Island: *Cylindrepomusbivitticollis* Breuning, 1947, *Cylindrepomuselisabethae* Hüdepohl, 1987, *Cylindrepomusperegrinussamarensis* Dillon & Dillon, 1948, and *Cylindrepomussexlineatus* Schultze, 1934. The most recent addition to the Philippine fauna is *Cylindrepomusnigerrimus* Vives, 2017 from northern Luzon.

The 2022 Philippine Coleopterological Expedition conducted by the Daugavpils University Beetle Research Team and the University of Mindanao Coleoptera Research Center yielded a diverse collection of beetles from different mountain ranges in Mindanao. Included in this collection was the new species of *Cylindrepomus* from northern Mindanao described herein, the fifth species of the genus known from Mindanao Island. An updated catalog of *Cylindrepomus* in the Philippines is included in this paper.

## ﻿Materials and methods

The new *Cylindrepomus* material was obtained during the 2022 Philippine Coleopterological Expedition through the Erasmus+ Mobility Programme of Daugavpils University in Latvia and the University of Mindanao Coleoptera Research Center, Philippines. The project aimed to document the coleopteran fauna in various mountain ranges in Mindanao, Philippines. The specimens under study were collected using hand nets along riparian fields at an elevation of approximately 600–700 m a.s.l.; specimens were killed with ethyl acetate. The habitat consists of an old-growth secondary forest with relatively high moisture and semi-open foliage allowing daylight to filter through.

Morphological characters were observed under Luxeo 4D and Nikon SMZ745T stereomicroscopes. Habitus images were taken with a Canon EOS 6D digital camera equipped with an MP-E macro lens. All images were then stacked using Helicon Focus and processed using a licensed version of the Photoshop CS6 Portable software.

Measurements of the various body parts follow Yoshitake and Yamasako (2016), with slight modifications concerning body length: **LB** = length of body from antennal support to apices of clothed elytra; **WH** = maximum width across head from the outer margin of a gena to that of another; **LG** = length of gena from upper margin to lower margin; **LL** = length of lower eye lobe from upper margin to lower margin; **WL** = maximum width across lower eye lobe; **LP** = length of pronotum from base to apex along midline; **WP** = maximum width across pronotum; **LE** = length of elytra from level of basal margins to apices of clothed elytra; **WEH** = width of elytra at humeri; / separates different lines on a label; // separates different labels. All measurements are given in millimeters.

Comparative materials and specimens used in this study are deposited in the following institutional collections:

**ANSP**Academy of Natural Sciences of Philadelphia, USA;

**MMCP** Milton Medina Collections, Mindanao, Philippines;

**MTKD** Senckenberg Naturhistorische Sammlungen Dresden, Germany;

**NRM**Naturhistoriska Riksmuseet, Stockholm, Sweden;

**SMF**Natur-Museum und Forschungs-Institut Senckenberg, Frankfurt am Main, Germany;

**UMCRC** University of Mindanao Coleoptera Research Center, Mindanao, Philippines;

**USNM**National Museum of Natural History (Smithsonian), Washington, D.C., USA;

**ZSM**Zoologische Staaatssamlung des Bayerischen Staates München, Germany.

## ﻿Catalog

***Cylindrepomusalbomaculatus* Breuning, 1947**

Arkiv för Zoologi, Uppsala, 39: p. 26; Breuning 1962: p. 417; Hüdepohl 1987: p. 74.

Distribution: Philippines.

Type and depository information: Holotype male, NRM.

***Cylindrepomusalbosignatus* Breuning, 1974**

Reichenbachia, Dresden, 15(5): p. 38; Hüdepohl 1987: p. 74.

Distribution: Philippines (Luzon: Panay, Gulasi, Zambales; Visayas: Mt. Macosolon in Capiz Western Visayas; Mindanao: Zamboanga).

Type and depository information: Holotype, MTKD.

***Cylindrepomusastyochus* Dillon & Dillon, 1948**

Transactions of the American Entomological Society, Philadelphia, 73: pp. 258, 262, pl. IX, fig. 14; Breuning 1962: p. 417; Hüdepohl 1987: p. 73.

Distribution: Philippines (Palawan; Visayas, Negros).

Type and depository information: Holotype male, ANSP.

***Cylindrepomusatropos* Dillon & Dillon, 1948**

Transactions of the American Entomological Society, Philadelphia, 73: pp. 257, 260, pl. IX, fig. 17; Breuning 1962: p. 416; Hüdepohl 1987: p. 73; Lingafelter et al. 2014: p. 21; Vives 2017: p. 52.

Distribution: Philippines (Luzon: Apayao; Visayas: Mt. Halcon in Mindoro, Samar).

Type and depository information: Holotype female, USNM.

***Cylindrepomusbayanii* Hüdepohl, 1987**

Entomologische Arbeiten aus dem Museum G. Frey, Tutzing bei München 35/36: pp. 74, 76, fig. 2.

Distribution: Philippines (Romblon).

Type and depository information: Holotype male, ZSM.

***Cylindrepomusbivitticollis* Breuning, 1947**

Arkiv för Zoologi, Uppsala, 39(6): p. 27; Breuning 1962: p. 416; Hüdepohl 1987: p. 74; Vives 2013: p. 72, fig. 12.

Distribution: Philippines (Mindanao: Mt. Kitanglad in Bukidnon).

Type and depository information: Holotype male, NRM.

***Cylindrepomuscicindeloides* Schwarzer, 1926**

Senckenbergiana, Frankfurt am Main, 8: p. 290, pl. 5, fig. 7; Breuning 1940: pp. 528, 537; Breuning 1962: p. 417; Hüdepohl 1987: p. 74.

Distribution: Philippines (Luzon: Mt. Banahao).

Type and depository information: Holotype, SMF.

***Cylindrepomuselisabethae* Hüdepohl, 1987**

**Figure 1.**
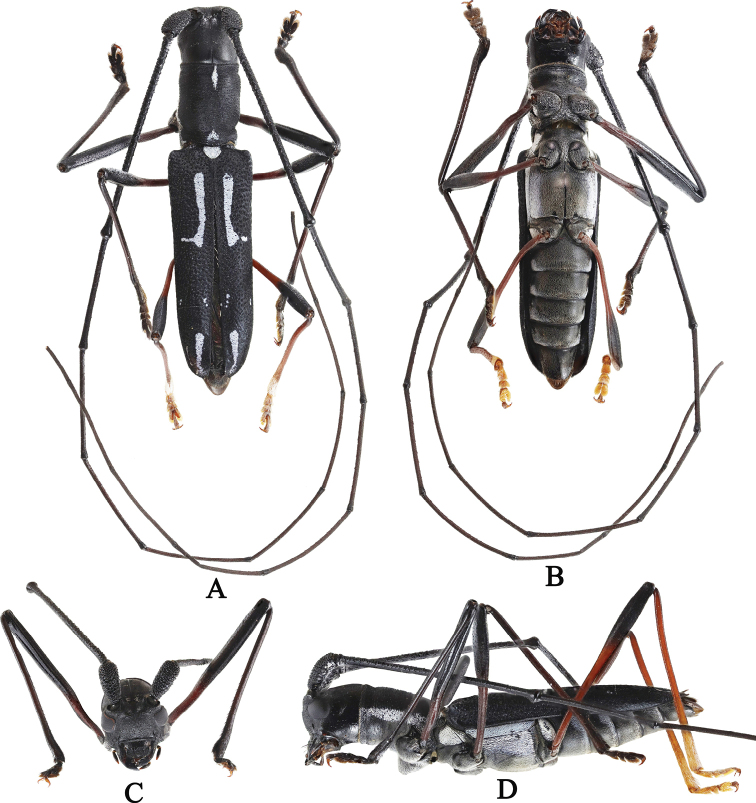
*Cylindrepomusansihagani* sp. nov. male holotype, habitus **A** dorsal aspect **B** ventral aspect **C** frons **D** lateral aspect.

Entomologische Arbeiten aus dem Museum G. Frey, Tutzing bei München 35/36: pp. 74–75, fig. 1.

Distribution: Philippines (Mindanao: Tandag Surigao del Sur).

Type and depository information: Holotype female, ZSM.

***Cylindrepomusflavicollis* Breuning, 1947**

Reichenbachia, Dresden, 15(5): pp. 25 ; Breuning 1962: p. 416; Hüdepohl 1987: p. 74.

Distribution: Philippines.

Type and depository information: Holotype male, NRM.

***Cylindrepomusmucronatus* Schwarzer, 1926**

Senckenbergiana, Frankfurt am Main, 8: p. 290, pl. 5, fig. 6 ; Breuning 1940: pp. 528, 537 ; Breuning 1962: p. 416; Hüdepohl 1987: p. 73.

Distribution: Philippines (Luzon: Imugan).

Type and depository information: Holotype male, SMF.

***Cylindrepomusnigerrimus* Vives, 2017**

Les Cahiers Magellanes, 25: p. 52, fig. 10.

Distribution: Philippines, Luzon, Nueva Vizcaya, Dupax del Sur.

Type and depository information: Holotype male, Collection E. Vives, Terrassa, Barcelona, Spain.

***Cylindrepomusperegrinussamarensis* Dillon & Dillon, 1948**

Transactions of the American Entomological Society, Philadelphia, 73: p. 264, pl. IX, fig. 10; Breuning 1962: p. 417; Hüdepohl 1987: p. 74; Lingafelter et al. 2014: p. 297.

Distribution: Philippines (Luzon: Panay; Visayas: Negros, Samar; Mindanao).

Type and depository information: Holotype male, USNM.

***Cylindrepomusrufofemoratus* Breuning, 1947**

Arkiv för Zoologi, Uppsala, 39(6): p. 47; Breuning 1962: p. 418; Hüdepohl 1987: p. 73.

Distribution: Philippines.

Type and depository information: Holotype male, NRM.

***Cylindrepomussexlineatus* Schultze, 1934**

The Philippine Journal of Science 53 (3): p. 312, pl. 1, fig. 3; Breuning 1940: pp. 529, 538; Breuning 1947: p. 6; Breuning 1950: p. 527; Breuning 1962: p. 416; Hüdepohl 1987: p. 74.

Distribution: Philippines (Mindanao: Lanao Province).

Type and depository information: Holotype female, MTKD.

Synonyms: *Cylindrepomussexlineatus* m. *ininterruptus* Breuning, 1950; *Cylindrepomussexlineatus* m. *reductevittatus* Breuning, 1947.

***Cylindrepomusysmaeli* Hüdepohl, 1987**

Entomologische Arbeiten aus dem Museum G. Frey, Tutzing bei München 35/36: pp. 74, 77, fig. 3.

Distribution: Philippines (Luzon: Mountain Province).

Type and depository information: Holotype female, ZSM.

## ﻿Taxonomy

### ﻿ Cylindrepomus ansihagani

Taxon classificationAnimaliaColeopteraCerambycidae

Medina & Cabras sp. nov.

38C3869C-4705-5F48-A027-28E050887B2E

https://zoobank.org/AF70CDCE-A5E7-445E-B5B0-FFA2AD50E42E

Fig. 1A–D

#### Holotype

(Fig. 1), male: Philippines – Mindanao / Northern Mindanao / Misamis Oriental III.2022 / local collector (MMCP), to be deposited at PNM.

#### Other material examined.

*Cylindrepomusbivitticollis* Breuning, 1947, holotype male, NRM; *C.sexlineatus* Schultze, 1934, holotype female, MTKD.

#### Description.

**Male.** Dimensions (*n* = 1): LB: 14.0 mm. WH: 2.0 mm. LG: 1.5 mm. LL: 1.0 mm. WL: 1.0 mm. LP: 3.0 mm. WP: 2.0 mm. LE: 8.5 mm. WEH: 3.0 mm.

***Teguments*** generally matt black, pro- and mesotibia reddish-brown near base; metatibia reddish-brown up to apical third; mid tarsus pale brown; hind tibia, tarsus, and claw light brown. Ventral side matt black, tomentose, covered with white recumbent pubescence on prosternum, prothorax, and abdomen.

***Head and gena*** tomentose, covered with black recumbent pubescence; genae with few erect black hairs at the side; vertex with two small bands of white recumbent pubescence. Eyes prominent, black, as long as wide. Antennae long and slender (except scape), more than twice the body length, matt black; scape bulbous, coarsely granulated, with recumbent white setae near base, 2× longer than wide; 2^nd^ antennomere wider than long; 3^rd^ antennomere coarsely granulated, 2× longer than each of antennomeres 4–11; 5^th^ antennomere slightly granulated; antennomeres 6–11 finely granulated.

***Pronotum*** 1.5× wider than long, tomentose, covered with black recumbent hairs; with two narrow bands of white recumbent pubescence, one at the base shaped like an elongated diamond, the other one triangle-shaped and near the margin; apical margin lined with golden setae.

***Prosternum*** tomentose, covered with black recumbent hairs at middle and white recumbent setae at sides. Mesosternum and metasternum tomentose, covered with black and white recumbent setae. Mesepisternum and metepisternum tomentose, covered with white recumbent setae. 1^st^ to 4^th^ abdominal ventrites tomentose, covered with white and black recumbent setae with sparse golden setae at each side; pygidium tomentose, covered with full black recumbent setae, apex lined with golden hairs (Fig. 1B).

***Elytra*** 2.5× longer than wide, with coarse, uniformly aligned punctatation; humeri slight recurved; suture and margin raised, slightly truncate along suture; apex lanceolate; with two thick bands of white recumbent pubescence, one at basal third longitudinal with apex expanded laterally, and one near the apex, narrowed toward the tip; a few tiny white spots near suture and margin at apical third. Scutellum tomentose, covered with white recumbent setae, obscuring surface (Fig. 1A).

Coxae tomentose, covered with whitish recumbent hairs; trochanters reddish-brown; tibia armed with two small spines at base (colored black on protibia and mesotibia, pale brown on metatibia). Profemur slightly recurved near base.

**Figure 2.**
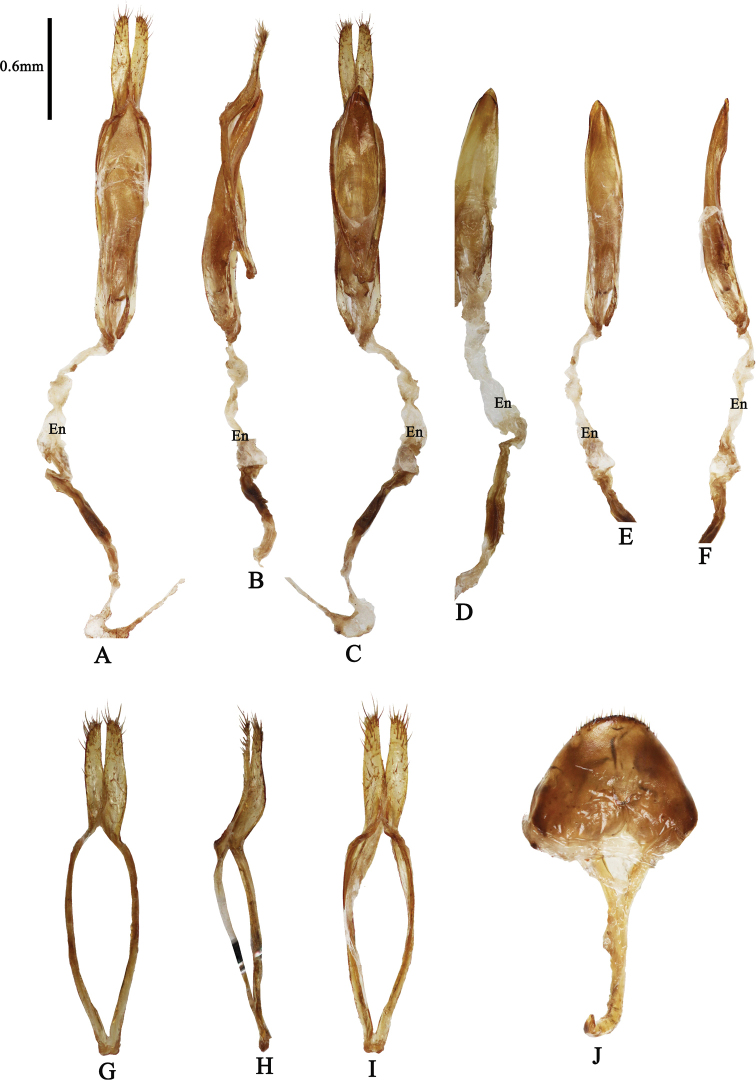
*Cylindrepomusansihagani* sp. nov. **A** genitalia, dorsal aspect **B** genitalia, lateral aspect **C** genitalia, ventral aspect **D** aedeagus, ventral aspect **E** aedeagus, dorsal aspect **F** aedeagus, lateral aspect **G** tegmen, dorsal aspect **H** tegmen, lateral aspect **I** tegmen, ventral aspect **J** tergite VIII. Abbreviation: **En** – Endophallus.

**Male genitalia** (Fig. 2 A–J): Tegmen ~1.5 mm long; lateral lobes slender, ~0.1 mm long and 0.6 mm wide; base with a broad central lobe bearing fine setae; apex bearing numerous golden setae, ~0.2–0.6 mm long. Aedeagus ~2.0 mm long and 0.5 mm wide, slightly recurved and tapering towards apex. Endophallus ~6.0 mm long.

#### Diagnosis.

*Cylindrepomusansihagani* sp. nov. is distinct from its Mindanao endemic congeners (*C.bivitticollis* and *C.sexlineatus*) in having pronotum with two narrow bands of white recumbent pubescence, one at the base, shaped like an elongated diamond, the other a triangular band of white recumbent pubescence near the margin, while *C.bivitticollis* has pronotum with a complete, pale yellow longitudinal band on each side of the disc and *C.sexlineatus* has pronotum with a yellowish spot on each side at the base.

#### Etymology.

This new species is named after Datu Ramil P. Ansihagan, the tribal chieftain of the Higaunon Tribe, for his efforts in protecting and preserving the remaining forests in Barangay Eureka Gingoog City, Philippines.

#### Distribution of *Cylindrepomusansihagani* sp. nov.

Philippines: Mindanao: Northern Mindanao, Gingoog City.

#### Notes on ecology, threats, and conservation of *Cylindrepomusansihagani* sp. nov.

The species is known from a single specimen that was collected during the expedition. The species was collected at an elevation of approximately 1000–1100 m a.s.l. using hand nets along the boundary between an agro-ecosystem and a secondary forest. Most of the trees present are endemic species including but not limited to *Shoreanegrosensis* (Red Lauan), *Shoreacontorta* (White Lauan), and *Quercussubsericea* (Philippine Ulayan Tree), all of which are native to the Philippines and considered highly valued trees.

The current threat to the species’ habitat is the continued conversion of the remaining secondary forests into agricultural lands. Farmers use various chemicals such as pesticides, herbicides, and fungicides that could affect the species’ population There is a need to conduct more expeditions, covering more habitats, to find additional populations of this and other species. Hence, research identifying the exact species distribution, area of occupancy and the species’ extent of occurrence is important as a guide in making a future IUCN Red List assessment of this endemic species.

## Supplementary Material

XML Treatment for Cylindrepomus ansihagani

## Supplementary materials

Supplementary material 1 Figure S1 Data type COL (jpg. image) Explanation note A catalog of the genus *Cylindrepomus* Blanchard, 1853 in the Philippines with description of a new species from Northern Mindanao is presented. Notes on the ecology, threats, and conservation of the new species is also provided. https://binary.pensoft.net/file/723414This dataset is made available under the Open Database License (http://opendatacommons.org/licenses/odbl/1.0/). The Open Database License (ODbL) is a license agreement intended to allow users to freely share, modify, and use this Dataset while maintaining this same freedom for others, provided that the original source and author(s) are credited.Milton Norman D. Medina, Melbert James G. Baul, Analyn A. Cabras
