# Supplementary figures and images for: ﻿Catalog of the genus Cylindrepomus Blanchard (Coleoptera, Cerambycidae, Dorcaschematini) in the Philippines, with description of a new species from northern Mindanao
Source: Zookeys. 2022 Aug 4;1116:23–32. doi: 10.3897/zookeys.1116.86906 (PMC9848631; doi:10.3897/zookeys.1116.86906)

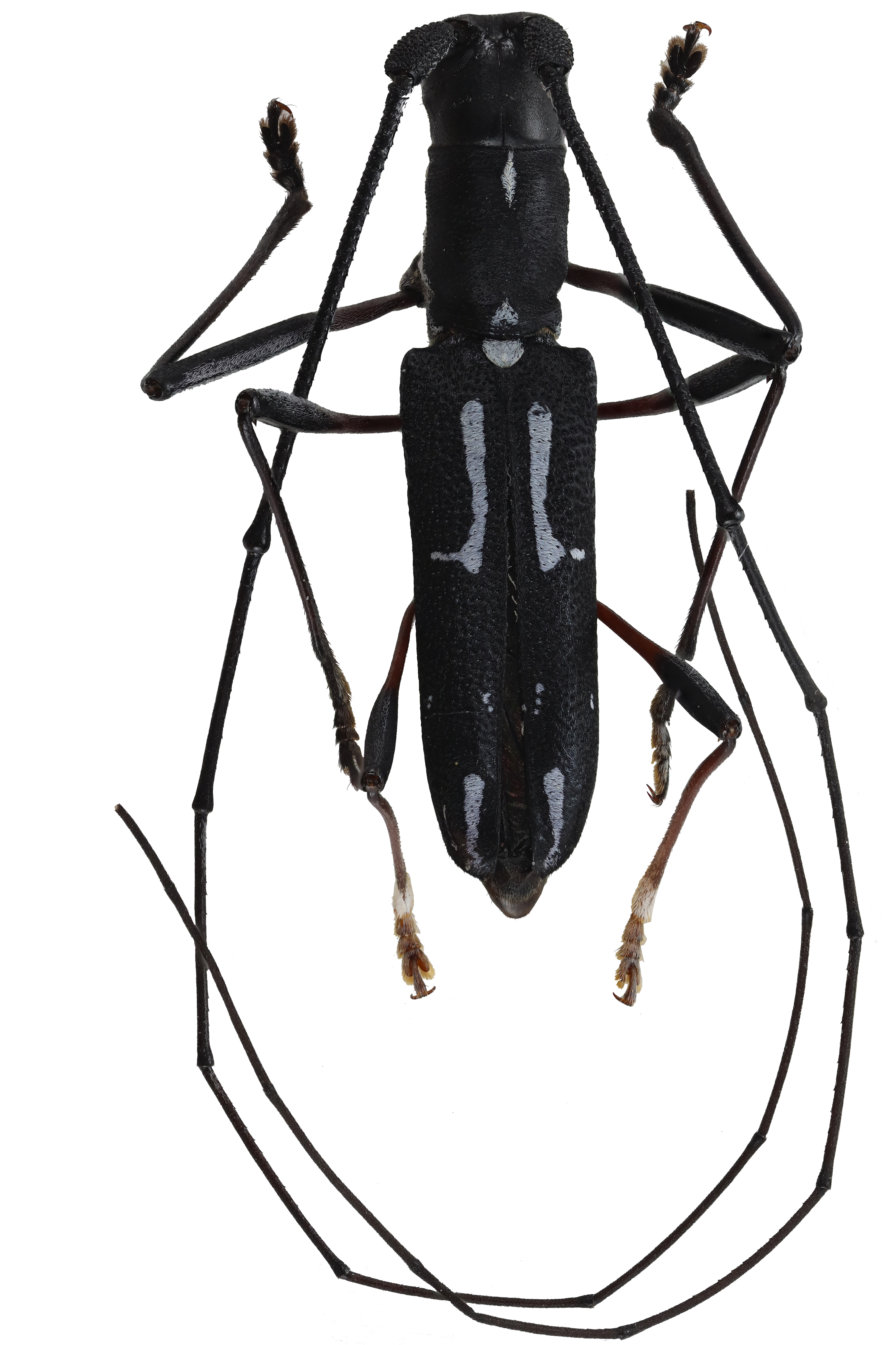

Supplement: Supplementary material 1 — Figure S1 [file zookeys-1116-023_article-86906__-s001.jpg]
